# Matrix: a complex amalgam of structures and functions in tumor microenvironment

**DOI:** 10.1002/2211-5463.70074

**Published:** 2025-06-21

**Authors:** Spyros S. Skandalis, Evgenia Tsoukala, Theodora D. Sarantopoulou, Maria‐Elpida Christopoulou

**Affiliations:** ^1^ Biochemistry, Biochemical Analysis & Matrix Pathobiology Res. Group, Laboratory of Biochemistry, Department of Chemistry University of Patras Greece; ^2^ Department of Pneumology Medical Center‐University of Freiburg, Faculty of Medicine, University of Freiburg Germany

**Keywords:** CD44, hyaluronan, integrins, matrix, proteoglycans, tumor

## Abstract

Cancer cells surrounded by a rich diversity of nonmalignant cell types are collectively embedded in the matrix, which is a dynamic, intricate three‐dimensional mesh of biomolecules with both structural and functional properties. The matrix contains proteins, carbohydrates, and other glycoproteins that facilitate essential cellular communication and impact in various ways a broad spectrum of cellular functions, such as anchoring cells, guiding migration, and shaping signal gradients driving cell growth, apoptosis, survival, and differentiation. This review deals with the complexity of this amalgam of structures and functions and highlights the importance of the tumor microenvironment in the maintenance and evolution of tumors by describing certain bioactive macromolecules of the matrix, such as proteoglycans, hyaluronan, collagens, elastin, matricellular proteins as well as their cellular receptors like integrins and CD44.

## Graphical abstract

The matrix is a dynamic, intricate three‐dimensional mesh of biomolecules with both structural and functional properties. This review deals with the complexity of this ‘molecular amalgam’ in the tumor microenvironment and highlights its importance in the maintenance and evolution of tumors by describing certain matrix biomolecules, such as proteoglycans, hyaluronan, collagens, elastin, matricellular proteins as well as specific matrix receptors.

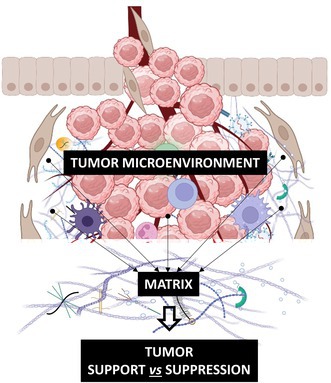

**Abbreviations**
ABC: ATP‐binding cassette
CAFs: cancer‐associated fibroblasts
CD44: cluster of differentiation 44
CD44s: CD44 standard isoform
CD44v: CD44 variant isoforms
CS: chondroitin sulfate
CSCs: cancer stem cells
CSPGs: chondroitin sulfate‐containing proteoglycans
DS: dermatan sulfate
DSPGs: dermatan sulfate‐containing proteoglycans
EBP: elastin‐binding protein
ECM: extracellular matrix
EDPs: elastin‐derived peptides
EMT: epithelial‐to‐mesenchymal transition
ERM: ezrin‐radixin‐moesin
GAG: glycosaminoglycan
HAS: hyaluronan synthase
Hep: heparin
HMW: high molecular weight
HS: heparan sulfate
HYAL: hyaluronidase
KS: keratan sulfate
LMW: low molecular weight
LOX: lysyl oxidase
LRRs: leucine‐rich repeats
MCPs: matricellular proteins
MDR: multidrug resistance
MMP: matrix metalloproteinase
NCAM: neural cell adhesion molecule
OPN: osteopontin
RGD: arginine‐glycine‐aspartate
RONS: reactive oxygen or nitrogen species
RTKs: receptor tyrosine kinases
SDC1: syndecan‐1
SLRPs: small leucine‐rich proteoglycans
SPARC: secreted protein acidic and rich in cysteine
SRGN: serglycin
TME: tumor microenvironment
TSP: thrombospondin
VCAN: versican

Tumor microenvironment (TME) is a highly structured ecosystem that contains tumor cells surrounded by a rich diversity of noncancerous cell types including cancer‐associated fibroblasts (CAFs), endothelial cells, immune cells, pericytes as well as tissue‐specific cells such as adipocytes and neurons [1]. All these cells are embedded in the extracellular matrix (ECM) or matrix, which is an intricate mesh of several macromolecules including fibrous proteins (collagens, elastin), glycoproteins (fibronectin, laminins), proteoglycans, hyaluronan, and matricellular proteins (Fig. 1). The matrix is produced, secreted, and remodeled by TME cells, and its specific composition is often decisive for tumor maintenance and metastatic potential. Moreover, tumors can gradually shape the tumor immune microenvironment into an immunosuppressive state to combat host immunity, and the balance between pro‐ and antitumor inflammatory matrix mediators may drive tumor progression. Importantly, the matrix facilitates essential intercellular communication by acting as a reservoir of diverse molecular effectors and substrates and, therefore, is a major determinant of TME properties. TME cells directly contact the surrounding matrix via their membrane receptors, like CD44 and integrins, coordinating the complex signaling networks that drive cancer [2, 3, 4]. Beyond its role as a structural framework that provides tissues and organs with mechanical stability, matrix influences essential processes such as branching morphogenesis, angiogenesis, stem cell niche formation, wound healing as well as congenital defects, tissue fibrosis, inflammatory disorders, and cancer. In this review, the roles of certain matrix bioactive molecules as constituents of this ‘amalgam’ of structures and functions within TME are discussed.

**Fig. 1:**
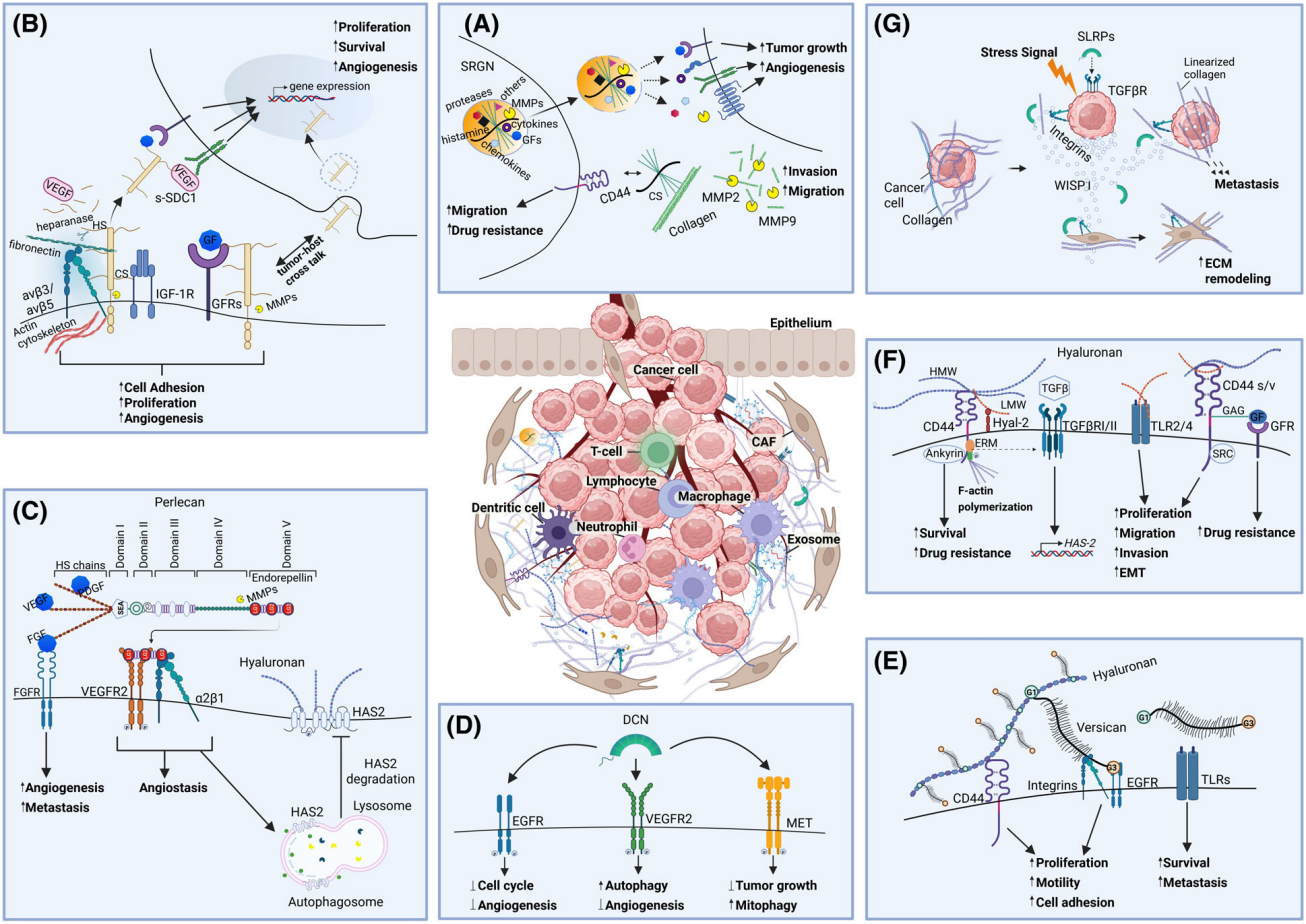
Structures, interactions, and functions of matrix biomolecules in the tumor microenvironment. (A) serglycin, (B) syndecan‐1, (C) perlecan, (D) decorin, (E) versican, (F) hyaluronan‐CD44, (G) WISP1. Please consult the manuscript for additional details. Image generated using BioRender.

## Proteoglycans

Proteoglycans are hybrid molecules that consist of a core protein onto which one or more glycosaminoglycan (GAG) chains are covalently linked while they are synthesized in the Golgi apparatus. These GAGs are negatively charged heteropolysaccharides containing repeating disaccharides of N‐acetylated hexosamines and hexuronic acid epimers forming chondroitin sulfate (CS), dermatan sulfate (DS), heparan sulfate (HS), heparin (Hep), and keratan sulfate (KS), which contains D‐galactose instead of hexuronic acid. Iozzo and Schaefer (2015) proposed a simplified and comprehensive classification of the various proteoglycan gene families and their products based on their cellular/subcellular topology, their gene/protein homology, and their specific core protein structural motifs [5]. According to their cellular/subcellular localization, proteoglycans are classified into four major classes moving from the cell interior to the extracellular space: intracellular/secretory, cell surface, pericellular and basement membrane zone and extracellular proteoglycans. The GAG substitution of proteoglycans seems to correlate with their topology and specific functions since heparan sulfate‐containing proteoglycans (HSPGs) predominate at the cell surface and the pericellular matrix, while chondroitin‐ and dermatan sulfate‐containing proteoglycans (CSPGs and DSPGs, respectively) are prevalent in the interstitial matrices [4, 5]. A major function of cell surface and pericellular HSPGs of great biological relevance is to bind and present growth factors (such as PDGF, HGF, VEGF, FGF) to their cognate cellular receptors thus critically regulating multiple aspects of signal transduction pathways as well as to generate long range maintenance of morphogen gradients during embryogenetic and regenerative processes. On the other hand, CSPGs and DSPGs function both as structural constituents of complex connective tissues/matrices (such as cartilage, tendons, brain, and eye) providing viscoelastic properties and proper matrix (i.e., collagen) organization as well as signaling molecules during tissue remodeling in pathological conditions like inflammation and cancer. Below, we discuss the structure and biological functions of certain proteoglycans from each class moving from the cell interior to the extracellular space in a centrifugal manner.

### Intracellular/secretory proteoglycans—Serglycin

Serglycin (SRGN) is an intracellular proteoglycan composed of a short core protein with eight Ser‐Gly residue repeats, where GAG chains are attached. The type and the sulfation pattern of these GAG chains (CS, DS, HS) vary depending on the cell type [6]. Notably, it is the only proteoglycan carrying Hep within the granules of mast cells and it aids in storing bioactive molecules such as growth factors, cytokines, and chemokines. Initially identified as a hematopoietic proteoglycan, SRGN is now known to be expressed by various cell types including tumor cells. It is presented in secretory intracellular compartments and, upon activation, is secreted into the ECM activating signaling pathways that regulate cell proliferation, migration, and inflammatory responses (Fig. 1A) [7, 8, 9].

Increased SRGN expression has been observed in aggressive tumor cells, including glioblastoma [10], breast cancer [11], nasopharyngeal carcinoma [12], multiple myeloma [13], and lung cancer [14]. During the exocytosis of vesicles, it increases the bioavailability of growth factors, such as VEGF and HGF, and cytokines, such as IL‐6, IL‐8, and CCL2, in the ECM promoting tumor growth and angiogenesis [8, 15, 16]. In multiple myeloma, SRGN provides a survival advantage for cancer cells, by binding to C1q/MBL and inhibiting complement pathways [17]. Additionally, the interaction with collagen I through its CS side chains provokes cellular invasion and migration by inducing the secretion of the matrix metalloproteinases MMP2 and MMP9, which participate in ECM remodeling [18]. CD44 contributes also to the pro‐oncogenic and pro‐metastatic functions of serglycin. SRGN has the ability to bind to CD44 via CS chains, followed by the activation of MAPK/β‐catenin and NF‐κB pathways, inducing migration and drug resistance [12, 19]. Research on gastric cancer cells has shown that CD44 increases the production of IL‐8 by CAFs, and in GCTB cancer cells, the SRGN/CD44/FAK system induces osteoclast differentiation from monocytes, supporting tumor progression [16, 20]. SRGN's presence in the TME from immune and stromal cells induces TGF‐β2 production via CD44/CREB1, which acts autocrinally by activating SMAD‐dependent pathways. This leads to the expression of epithelial‐to‐mesenchymal transition (EMT)‐related transcription factors (ZEB1, SNAIL), driving metastasis and self‐renewal [10, 21]. In contrast to the promotional role of serglycin in cancer, recent results in breast cancer cells show low expression of the molecule compared to normal tissue. The levels of serglycin in this case are likely attributed to immune and myeloid cells, requiring further studies to determine the exact molecular mechanisms in each cancer type [21].

### Cell surface proteoglycans

This class contains seven transmembrane proteoglycans (syndecans 1–4, CSPG4/NG2, betaglycan/TGF‐β type III receptor, phosphacan/receptor‐type protein tyrosine phosphatase β) and six GPI‐anchored glypicans (glypicans 1–6) [5]. We will focus to syndecans, in particular syndecan‐1, which are involved in multiple biological functions in normal development and perturbed states including cancer.

#### Syndecan‐1

Syndecan‐1 (SDC1) is the first studied member of the syndecan family of transmembrane HSPGs. Its structure is defined by an extracellular region that carries HS and CS chains, a single transmembrane domain and a well‐conserved cytoplasmic domain (Fig. 1B) [22]. In addition to its critical role in maintaining homeostasis, SDC1 is involved in the development and progression of various pathological conditions, including cancer [23]. SDC1 acts as a coreceptor by binding growth factors, such as FGF, VEGF, PDGF, HGF, TGF‐β, and Wnt, through its HS chains to stabilize the ligand/receptor complexes. This is followed by the activation of downstream signaling pathways such as MAPK, PI3K/AKT, and β‐catenin, which promote cell proliferation, angiogenesis, and the survival of cancer cells [24, 25, 26, 27]. The protein core of the SDC1 ectodomain has been found to bind to integrins αvβ3/αvβ5, while forming a trimeric complex with IGF1‐R, enhancing angiogenesis and cell adhesion. In this way, the reduced expression of SDC1 affects cell motility and promotes EMT [28, 29]. Due to its C1 and C2 cytoplasmic structural domains, SDC1 interacts with actin‐related proteins or molecules containing PDZ domains, thereby regulating cytoskeletal rearrangement and the trafficking of endosomes and exosomes [26].

Apart from being located on the cell surface, SDC1 exists also in a soluble form (s‐SDC1) (Fig. 1B). The extracellular domain, along with its GAG chains, is shed from the membrane through MMP‐mediated proteolysis, released into ECM, and enters the circulation [23]. Studies on breast and myeloma cancer cells have shown that soluble SDC1 interacts with growth factors similarly to the intact membrane‐bound molecule promoting paracrine interactions and influencing cell proliferation and angiogenesis. It exhibits both competitive activity, reducing the signaling that promotes tumor growth, and cooperative activity by promoting signal transduction [24, 30]. Additionally, binding to integrins on endothelial cells promotes the development of a mesenchymal phenotype [31]. Cleavage of SDC1 promotes SUMOylation of AKT highlighting a role of PI3K/AKT in SDC1‐mediated breast cancer proliferation [30]. Interestingly, myeloma tumor cell‐derived shed SDC1 is uptaken by bone marrow stromal cells and is translocated to the nucleus, in an HS‐dependent manner, where it binds and inhibits histone acetyltransferase (HAT) p300 providing a mechanism for tumor–host cell crosstalk [32, 33].

The role of SDC1 is altered during tumorigenesis in different cancer types. In breast cancer, increased expression of SDC1 on the cell surface was associated with reduced overall survival in ER‐ cells, while higher levels of stromal SDC1 indicate poor survival prognosis for patients with ER+ cancer. In lung cancer cells, high SDC1 expression was linked to a prediction of invasiveness but favorable survival after surgery, whereas elevated soluble SDC1 indicated poor clinical outcome. On the other hand, decreased epithelial expression of SDC1 showed tumor aggressiveness in head and neck cancer cells [24, 30]. Therefore, the expression pattern of SDC1 in the TME varies depending on its localization and tissue of origin while it is not yet clear if its increased or decreased expression indicates a more positive prognosis.

### Pericellular and basement membrane zone proteoglycans

Pericellular proteoglycans include perlecan, agrin, and collagens XVIII/XV [5]. These four proteoglycans carry mostly HS side chains and create a pericellular plasma membrane‐associated negatively charged layer that binds and regulates the availability and activity of various extracellular ligands such as basic growth factors. Perlecan is a prominent member of this group of proteoglycans.

#### Perlecan

Perlecan is a HSPG that is a key component of basement membranes. It is expressed in both vascular and avascular tissues, participating in crucial biological processes and it consists of five distinct domains (Fig. 1C) [5, 34]. The N‐terminal domain (domain I) of perlecan, primarily carrying three HS chains, includes homology with sea urchin sperm protein (SEA), which has increased diversity for interacting ligands, such as laminin, collagen IV and XI, nidogen, TSP‐1, PRELPs, and growth factors. Domains II, III, and IV are homologous to regions in LDL receptors, laminin B, IgG, and neural cell adhesion molecule (NCAM), respectively [34, 35]. Perlecan's significance in cancer progression has been confirmed by various studies on breast [36], prostate [37], colon [38], oral squamous [39], and pancreatic [40] cancers. It appears as a pro‐angiogenic factor, promoting tumor growth, migration, and invasion and also contributes to drug resistance. This tumorigenesis‐promoting activity is mainly due to interaction with heparin‐binding growth factors, including FGF, VEGF, PDGF, HGF, and Hedgehog, which induce angiogenesis and metastatic spreading [41, 42].

In contrast, the C‐terminal fragment of perlecan (domain V), known as endorepellin, is released from the endothelial basement membrane via MMPs and has angiostatic and antitumorigenic effects (Fig. 1C) [43]. Endorepellin comprises of three laminin‐like globular (LG) regions of which LG1/2 bind to VEGFR2 and LG3 concurrently binds to the α2 subunit of α2β1. Through these interactions, endorepellin signals as a VEGFA antagonist, inhibiting pro‐angiogenic signaling pathways [44]. Also, it has been found that it activates AMPK and PERK/eIF2a/ATF4/GADD45a pathways, promoting autophagy and mitophagy in endothelial cells [45, 46, 47]. Research findings from breast cancer cells indicate that endorepellin is also involved in autophagosomal degradation of HAS2, reducing extracellular hyaluronan in TME, which limits cell proliferation and angiogenesis [48, 49]. Consequently, the dual nature of perlecan which exerts pro‐angiogenic functions in its intact form while its degradation leads to the liberation of bioactive fragments displaying potent angiostatic activity reflects the multifaceted, complex and even opposing nature not only of the extracellular matrix as a whole but also of single matrix molecules.

### Extracellular proteoglycans

Extracellular proteoglycans include the following: four hyalectans (aggrecan, versican brevican, and neurocan), an acronym for hyaluronan‐ and lectin‐binding proteoglycans that form supramolecular complexes of high viscosity and act as molecular bridges between cell surfaces and ECM; eighteen small leucine‐rich proteoglycans (SLRPs) that are characterized by a relatively small core protein with a central region constituted by leucine‐rich repeats (LRRs) grouped into five classes (I‐V) (decorin, biglycan, lumican, fibromodulin, PRELP, and others); and the testican/SPOCK family of proteoglycans that comprise three members [5]. Due to lack of space, we will refer to decorin (SLRP) and versican (hyalectan), two extracellular proteoglycans with often contradicting functions in tumor biology.

#### Decorin

Decorin, the prototype member of the SLRP family, is expressed in most tissues mainly in stromal and endothelial cells. It consists of a central curved domain rich in leucine residues responsible for the interaction with collagen, flanked by terminal sequences rich in Cys and attached with one N‐terminal GAG chain (CS or DS) (Fig. 1D). In addition to the ability of collagen fibrils ‘decoration’, decorin interacts with ECM components, growth factors, and cell surface receptors, contributing to various cellular functions that regulate the cell cycle, inflammation, fibrosis, autophagy, and have crucial roles in cancer [5, 50, 51, 52].

Τhe presence of decorin in tumor stroma impedes the tumorigenic process [52, 53]. Indeed, studies from decorin‐deficient mice showed increased cellular proliferation and induced EMT [54]. Mechanistically, decorin binds TGF‐β, particularly the TGF‐β1 isoform, and PDGF preventing binding to their cognate receptors and suppressing downstream signaling pathways [55, 56]. This PG acts as a global antagonistic ligand of receptor tyrosine kinases (RTKs) downregulating signaling pathways that drive angiogenesis and invasion in tumor microenvironment (Fig. 1D) [57]. Several studies on breast cancer [58, 59], and epidermoid carcinoma [60] proved that decorin by binding to EGFR blocks PI3K and Ras signaling responsible for sustained tumor growth and activates anti‐angiogenic MAPK pathway in order to enhance the expression of cyclin inhibitor p21^waf^ and thrombospondin‐1 (TSP‐1) [51]. Decorin also destabilizes E‐cadherin via EGFR/ERK signaling, suppressing tumor cell invasion and metastasis [58]. Moreover, decorin promotes EGFR endocytosis into caveolin‐1 vesicles and degradation in lysosomes, followed by Ca^2+^ release intracellularly [61]. By interacting with VEGFRs (VEGFR2, VEGFR3) and IGF‐IR, decorin evokes autophagy in endothelial cells, inhibiting mTOR and activating Peg3 for the formation of autophagosomes [62]. In carcinoma and vascular endothelial cells, it has been found that decorin is attached to Met receptor (HGFR) and via mitostatin, causes mitochondrial depolarization and mitophagy [63, 64]. Binding of decorin to Met induces its transient activation and c‐Cbl ubiquitin ligase recruitment resulting in rapid Met proteasomal degradation and suppression of β‐catenin intracellular levels thus inhibiting Met‐driven tumor cell growth and migration [65]. Overall, the multifaceted capacity of decorin to attenuate *in vivo* tumor growth and metastatic spreading has long been well‐established. Several studies confirm that reduced expression of decorin in tumor stroma is associated with poor prognosis in metastatic invasive breast cancer and complete absence promotes the progression of several tumor types, including urothelial carcinoma, squamous cell carcinoma, and myeloma [56].

#### Versican

Versican (VCAN) is a large CS/DSPG that belongs to the hyalectans [5, 66]. VCAN isoforms (V0, V1, V2, V3, V4), which arise from alternative splicing of the main exons encoding the GAG‐binding regions (GAGα and GAGβ), are expressed in various tissues [67]. They have the ability to interact with molecules of the ECM and cell surface receptors, with crucial roles in inflammatory conditions and cancer (Fig. 1E) [68]. Increased expression of VCAN has been found in the TME in multiple cancers, such as breast [69], colon [70], lung [71], and liver [72], gastric [73, 74] cancers, contributing to tumor cell proliferation, metastasis, and resistance to apoptosis [75]. The N‐terminal G1 domain of VCAN, which includes an immunoglobulin (Ig) homolog module and two hyaluronan‐binding domains, binds to hyaluronan and interacts with CD44, stimulating tumor spreading, self‐renewal, and angiogenesis, while reducing matrix permeability. On the other hand, the hyaluronan and VCAN‐rich matrix inhibits the spreading and migration of immune cells, protecting cancer cells from immune responses [68, 75]. Besides CD44, VCAN binds to TLRs, promoting the secretion of pro‐inflammatory cytokines (TNF‐α, IL‐6) that prevent cancer cell apoptosis and enhance metastasis [76]. Furthermore, studies on CAFs and CSCs demonstrate that VCAN expression is regulated by TGF‐β and the transcription factor SNAIL, inducing EMT and invasion [77, 78, 79, 80]. The C‐terminal G3 region of VCAN, with two structural repeats resembling EGF (EGF‐like), activates the EGFR/AKT/GSK‐3β signaling pathway and augments tumorigenesis and resistance to chemotherapeutic drugs [75, 81]. VCAN's fragments after regulated proteolysis by ADAMTS and MMPs, known as VCAN‐matrikines, have supporting roles in these diverse cancer‐promoting processes [66].

## Hyaluronan

Although it has almost been a century since the first official report of hyaluronan in 1934 by Karl Meyer and John W. Palmer, there is still an emerging ongoing survey on the profound comprehension of the role that hyaluronan polysaccharide poses in both physiological and pathological events, primarily focusing on cancer progression (Fig. 1F) [82, 83, 84, 85, 86]. Hyaluronan is a common component of the different ECMs in vertebrate connective tissues, mainly in cartilage, skin, brain, vitreous body, umbilical cord, and synovial fluid [87]. The structural stone of this nonsulfated unbranched GAG is the disaccharide unit of D‐glucuronic acid and N‐acetyl‐D‐glucosamine linked by alternate β (1➔3) and β (1➔4) glycosidic bonds. In contrast to the other GAGs, the hyaluronan biosynthesis occurs at the plasma membrane, rather than the Golgi apparatus, by specific hyaluronan synthase isoenzymes (HAS 1, 2, and 3) [88]. As additional units are incorporated, the resultant hyaluronan polymer attains a progressively higher molecular weight and is exported to the pericellular and extracellular spaces. Notably, under certain developmental or pathological states, hyaluronan is also located within cells regulating vital cellular processes like mitosis, cell cycle progression, RNA processing, and autophagy [89].

The equilibrium between the synthesis and the breakdown of hyaluronan by the coordinated actions of HASes and its degrading enzymes (mainly HYAL1, HYAL2, PH‐20, KIAA1199, and TMEM2) may be the key to understanding how normal functions turn into malignant ones based on the interactions that hyaluronan has with different molecules of the matrix and specifically the diverse behavior of the amount and size of hyaluronan molecules [82, 84, 90, 91, 92]. The functions that require proper hyaluronan concentration range from embryogenesis and tissue development to the homeostasis of the immune system, thus deregulation of HASes—due to multiple mechanisms—leads to abnormal distribution of hyaluronan in the tissues both qualitatively and quantitatively. Hyaluronan of various sizes accumulates in a greater ratio in malignant tissues than in the corresponding healthy ones due to the imbalanced activity of HASes, hyaluronan‐degrading enzymes and reactive oxygen or nitrogen species (RONS) [93, 94, 95, 96, 97, 98, 99, 100, 101, 102, 103]. Long now it has been established that stromal cells are responsible for hyaluronan production in the TME as stimuli by factors that tumor cells release, along with tumor cells themselves, infiltrating immune cells, CAFs, and several other TME cell types [86, 104, 105, 106, 107]. The interactions between hyaluronan and hyaladherins (such as CD44, hyalectans, and TSG‐6) lead to ECM remodeling, thus alternating normal metabolic pathways of the cells [3, 108, 109]. The role of hyaluronan can differ depending on the type of cancer, with some tumors showing more pronounced hyaluronan involvement than others. Both the amount and molecular weight play pivotal roles, as the effect of hyaluronan can be either anti‐ or pro‐oncogenic. Generally, high molecular weight (HMW)‐hyaluronan is a predominant component in the ECMs of healthy tissues due to its peculiar physicochemical and scavenging properties; on the contrary, hyaluronan fragmentation by the hyaluronan‐degrading enzymes and/or RONS leads to low molecular weight (LMW)‐hyaluronan molecules that have been shown to promote tumor growth, cancer cells stemness, metastasis, resistance to chemotherapy, and tumor‐associated immune suppression [91, 106, 110, 111, 112, 113]. Amorin et al. demonstrated that gastric cancer cells attached to hyaluronan oligomers (6.4 kDa) develop an aggressive phenotype resulting in high invasiveness whereas in surfaces with HMW‐hyaluronan, the cells were not prone to migration due to the activation of the p38 pathway aside from ERK1/2 inhibition [102]. Even though LMW‐hyaluronan is mainly accused of carcinogenesis, new evidence about HMW‐hyaluronan proposes that the nonfragmented hyaluronan can facilitate tumor resistance by shaping its immune microenvironment, a procedure way different than its casual role as a lubricant with anti‐angiogenic function in the intermediate space between endothelial cells [114, 115, 116, 117]. The interaction of LMW‐hyaluronan with Toll‐like receptors, especially TLR2 and TLR4, has been accused of cancer cell proliferation and the suppressive immune system of the TME. In breast cancer, the binding of LMW‐hyaluronan with CD44 and TLR2/4 promotes cell invasion, along with the activation of NF‐κB pathway and transcription and release of pro‐inflammatory cytokines such as ΤΝF‐α, IL‐1β, IL‐8, and IL‐12. Such conditions lead to metastasis and malignancy progression, as in the case of melanoma (reviewed in [118]). Another fact proving the tumorigenic behavior of hyaluronan is its contribution to EMT, promoting pro‐metastatic properties alongside cell plasticity and resistance in conventional chemotherapy. It has been demonstrated that HAS2 is required for the induction of the EMT program mediated by TGF‐β specifically in breast cancer cells coupled with the participation of hyaluronan receptor CD44 [119, 120]. Another feature in a cancerous ECM that leads to tumor drug resistance is that hyaluronan controls multidrug resistance (MDR) mechanisms by influencing cell membrane transporters of the ATP‐binding cassette (ABC) [113]. Often during therapy, a remarkable drug efflux is observed due to the upregulated function of such transporters by hyaluronan intermediated action, while hyaluronan can also obstruct drug delivery to the desired area by preventing blood vessel function. A rise in interstitial fluid pressure causes vascular collapse thus there are significant obstacles to the perfusion, diffusion, and convection of small‐molecule drugs [113, 121, 122]. In the era of personalized medicine, it is of great importance to develop targeted therapy based on each patient's malignancy, effective and without side effects, given that cancer is a very heterogeneous condition. Accumulation of hyaluronan together with increased synthesis of CD44 and specific HYALs correlate with tumor progression and reduced overall patient survival in certain types of tumors. Therefore, inhibition of hyaluronan may be a potent matrix‐based targeted treatment of metastatic tumors like triple‐negative breast cancer [123, 124, 125]. On the other hand, hyaluronan seems to be a handy contributor to this effort as it is naturally found in healthy tissues and can easily be modified. For example, sulfated hyaluronan is a promising modified polymer with a great range of actions including anticancer and pro‐osteogenic effects, inhibition of angiogenesis and hyaluronan‐degrading enzymes, promotion of wound healing, drug delivery agency, and bioengineering applications [116, 126].

## Collagens and elastin

Collagens and elastin are essential structural components of the ECM, providing mechanical support and elasticity to tissues [127]. These proteins contribute to tissue architecture and regulate cellular behaviors such as adhesion, migration, and differentiation [128]. Collagens, which form a family of ECM proteins with a characteristic triple‐helix structure, include 28 distinct types (I–XXVIII) [129, 130]. They are vital for ECM organization and significantly influence the mechanical properties of tissues [127, 131]. They form scaffolds that ensure structural integrity and interact with cell surface receptors, further affecting cellular behaviors [3]. Elastin, on the other hand, is an insoluble biopolymer, consisting of units of its soluble precursor, tropoelastin [132, 133]. Tropoelastin comprises interchanging hydrophilic and hydrophobic regions, which are encoded by different exons, each with specific functions [134]. The hydrophilic domains, featuring motifs such as lysyl‐alanine (KA) and lysyl‐proline (KP), drive cross‐linking and elastin maturation through the activity of lysyl oxidase (LOX) and LOXL enzymes [135]. By contrast, hydrophobic regions confer elasticity and play a vital role in promoting cellular interactions and maintaining tissue flexibility [136]. Both collagen and elastin contribute to a complex, interconnected ECM network that supports carcinogenesis [137]. Collagen deposition, primarily by CAFs, increases ECM stiffness, which is associated with enhanced migration, invasion, and metastasis of cancer cells [138]. This mechanical alteration affects integrin‐mediated signaling by activating FAK pathways that promote cancer cell motility [139, 140]. In addition, LOXs, which cross‐link collagen fibers, further harden the ECM and support processes such as EMT, a key mechanism for metastasis [141, 142]. Additionally, collagen degradation by MMPs produces bioactive peptides known as matrikines [143]. These collagen‐derived matrikines, such as fragments from collagen type IV (e.g., tumstatin), bind to integrins (αvβ3, α5β1) and inhibit the FAK/PI3K/Akt/mTORC1 pathway, reducing cell proliferation and invasion [144], while fragments from collagen type I (e.g., proline–glycine–proline or PGP) enhance neutrophil recruitment and inflammation, further shaping the TME. Other matrikines may promote tumor progression by activating different integrin receptors and pathways. Furthermore, elastase‐mediated elastin degradation, releasing elastin‐derived peptides (EDPs) act as bioactive signaling molecules that modulate the TME [145]. EDPs, such those containing GxxPG motif, interact with elastin‐binding protein (EBP) and integrins to activate signaling pathways that regulate angiogenesis, immune evasion, and cancer cell motility [146, 147]. Moreover, EDPs may activate MMPs and inflammatory cytokines, perpetuating ECM remodeling and facilitating tumor invasion [148, 149, 150, 151]. These synergistic effects of collagen and elastin within the ‘matrix amalgam’ orchestrate the mechanical and biochemical environment that supports tumor progression.

## Matricellular proteins

Matricellular proteins (MCPs) are a group of nonstructural ECM molecules that play key roles in regulating cellular behavior, particularly in the context of cancer and the tumor microenvironment [152]. Unlike structural ECM proteins such as collagen and elastin, which provide mechanical support and form physical structures like fibrils and basal laminae, MCPs modulate cellular functions through interactions with cell surface receptors, proteases, hormones, and other ECM components. These proteins influence a variety of biological processes such as tissue remodeling, inflammation, and survival while supporting cancer stem cells (CSCs), which drive tumor initiation and metastasis [153, 154].

Key matricellular proteins—thrombospondins (TSP1, TSP2), SPARC (secreted protein acidic and rich in cysteine), osteonectin, and tenascins—modulate critical processes in cancer and the TME. Among these, thrombospondins and SPARC have garnered particular attention for their dual roles in tumor suppression and progression. For instance, TSP2 binds MMP2 to suppress tumor growth and angiogenesis but also enhances the migratory potential in osteosarcoma by upregulating MMP9 expression [155, 156]. Similarly, SPARC promotes cell migration by repressing E‐cadherin and activating integrin αvβ3 [157] and regulates collagen fibril organization and adhesion by mediating procollagen I processing and its incorporation into the ECM [158]. Tenascin‐C, on the other hand, promotes cell migration in aggressive cancers by interfering with fibronectin‐syndecan‐4 binding [159]. Syndecans and CD44, modulated by MCPs like SPARC and osteopontin (OPN), further mediate ECM signaling, with syndecans acting as coreceptors for growth factors, like VEGF, and CD44 promoting EMT, invasion, and metastasis through hyaluronan binding [160].

Other notable families of MCPs include CCNs, fasciclin, galectins, R‐spondins (RSPOs), fibulins, SIBLINGs, and olfactomedins [161]. Among these, the CCN family stands out as a prominent subgroup of secreted, multi‐modular, cysteine‐rich proteins that play crucial roles in cell communication, tissue dynamics, and the regulation of cellular processes. The CCN family consists of six members: Cyr61 (CCN1), CTGF (CCN2), NOV (CCN3), WISP1 (CCN4), WISP2 (CCN5), and WISP3 (CCN6) [162].

Within the CCN family, WISP1 (CCN4) has emerged as a particularly intriguing protein due to its critical involvement in tumor biology and Wnt‐1‐induced signaling pathways [163]. Distinguished by its multi‐modular structure, comprising several functional domains (IGFBP, VWC, TSR, and CT regions), WISP1 interacts with ECM components, cell surface receptors, and signaling molecules in both autocrine and paracrine manners (Fig. 1G). Such interactions allow WISP1 to modulate cellular behavior within the TME, influencing processes like cell adhesion, migration, and survival [164]. In particular, WISP1 binds to collagens, fibronectin, and SLRPs like decorin and biglycan, altering the matrix composition and rigidity [165, 166]. These interactions impact matrix stiffness and cell adhesion while modulating key signaling pathways that facilitate tumor invasion. Notably, WISP1 promotes type I collagen linearization, a hallmark of aggressive tumors, by directly binding collagen and enhancing tumor invasion and metastasis independent of cell‐generated mechanical tension [167]. Elevated WISP1 expression correlates with faster metastatic progression and poor prognosis, although CAF‐derived WISP1 has been shown to inhibit lung metastasis [168]. Furthermore, WISP1 exacerbates tumor progression by enhancing EMT driving tumor cell migration and metastasis [169]. Additionally, WISP1 contributes to angiogenesis by modulating endothelial cell behavior and interacting with pro‐angiogenic factors such as VEGF, thereby promoting new blood vessel formation necessary for tumor growth [170]. Beyond its structural and angiogenic roles, WISP1 influences immune cell dynamics within the TME, regulating immune cell recruitment and activation to favor tumor progression [164, 171]. Moreover, WISP1 activates critical signaling pathways, including PI3K/AKT and β‐catenin, promoting tumor cell proliferation, survival, and resistance to apoptosis [172].

Overall, the diverse functions of MCPs within the tumor microenvironment highlight their critical role in regulating cancer progression and metastasis; however, WISP1's unique contributions—such as its role in collagen linearization, immune modulation, and angiogenesis—underscore its potential as a novel therapeutic target.

## Matrix receptors

Cells transduce extracellular signals into intracellular responses through their transmembrane receptors. Among these receptors are integrins, syndecans, discoidin domain receptors, and cluster of differentiation 44 or CD44, which are the main matrix receptors.

### CD44

CD44 operates as a receptor for various matrix molecules including OPN, collagen, fibronectin, laminin, growth factors, cytokines, MMPs, SRGN and, primarily, hyaluronan (Fig. 1F) [173]. CD44 is a single‐chain transmembrane glycoprotein or part‐time proteoglycan (under certain conditions) that is encoded by a single gene containing constant and variant exons. Alternative splicing of the 10 or 9 variant exons in mice and humans, respectively, generates multiple CD44 variants (CD44v) contributing to the complexity of the stem region of CD44 proteins [3, 174]. CD44v show a more specialized expression pattern than the ubiquitously expressed standard form of CD44 (CD44s; encoded by the constant exons) [175]. Specific CD44v are associated with the progression and stemness of various tumors, including breast [176], prostate [177], colorectal [178, 179], pancreatic [180], and glioma [181, 182]. However, CD44s expression is also correlated to the aggressiveness of specific tumors since it has been reported that CD44s but not CD44v induces tumor initiation and CSCs gene traits through PDGFRβ‐STAT3 axis [183]. Similarly, CD44 splice isoform switching from CD44v to CD44s was essential for EMT and tumor metastasis [184], while the interaction of phosphorylated cortactin with CD44s but not CD44v induced the activation of invadopodia driving tumor cell invasion and metastasis [185]. Of particular interest is the great potential of the short intracellular domain, common to all CD44 proteins, to interact with various cytoplasmic proteins, such as ezrin‐radixin‐moesin (ERM), ankyrin, Src kinases and Oct4/Sox2/Nanog, thus regulating the cell‐trafficking machinery, cell shape and morphology, transcriptome, cell metabolism and multiple signal transduction pathways [186, 187, 188, 189, 190]. Importantly, CD44 is part of the CSC signature in several tumor types, while hyaluronan‐CD44 axis is critically involved in the maintenance of stemness and multidrug resistance and, thus, its targeting could constitute a potent and efficient approach to restrain stemness properties and prevent disease relapse [85, 191, 192, 193].

### Integrins

Integrins are heterodimeric transmembrane receptors that mediate cell–cell and cell–matrix adhesion. Different combinations of 18 α‐subunits with 8 β‐subunits result in the formation of at least 24 heterodimeric integrins that have varying affinities to extracellular ligands and are clustered into arginine‐glycine‐aspartate (RGD)‐binding integrins, collagen‐binding integrins, laminin‐binding integrins, and leukocyte‐specific integrins. Integrins can carry out both outside‐in and inside‐out signaling pathways thus regulating cell polarity, survival, cytoskeleton rearrangement as well as cell adhesion and migration [3, 194]. Integrins are actively involved in many of the steps of cancer progression from primary tumor formation to cancer cell extravasation and formation of a metastatic niche. Upregulation and clustering of integrins result in increased outside‐in signaling by activating FAK, Src, PI3K‐Akt, and MAPK followed by increased cancer cell migration, protease activation, ECM stiffness, anchorage‐independent survival, and metastasis [194, 195]. Integrin signaling is not restricted to the plasma membrane but also unconventional modes of integrin signaling exist contributing to cancer cell survival, stemness, and drug resistance. For example, α6β1 and α6β4 integrins in tumor exosomes appear to target metastatic cells to the lung, while αvβ5 integrin to the liver by preparing the suitable premetastatic niche through the specific overexpression of laminin and phosphorylated Src or fibronectin in these target tissues for the establishment of metastatic tumors [196, 197, 198, 199].

## Conclusive remarks

Matrix influences essential processes such as stem cell niche formation, branching morphogenesis, angiogenesis, bone remodeling, and wound healing, as well as pathological conditions like tissue fibrosis and cancer. There is a growing number of examples showing that individual matrix components can significantly affect disease phenotypes [200]. Consequently, targeting or utilizing these components or their synthetic analogs (mimetics) holds promise for therapeutic intervention. For instance, in tumors with high levels of hyaluronan, such as pancreatic ductal adenocarcinoma, the dense ECM can act as a physical barrier to drug delivery. The use of PEGylated hyaluronidase (PEGPH20) to enzymatically degrade hyaluronan has been shown to improve the intratumoral penetration of chemotherapeutic agents, illustrating how modulating matrix composition can directly enhance treatment efficacy [201]. Another example involves integrins, key matrix receptors that regulate cell adhesion, migration, and survival. The therapeutic peptide cilengitide, which targets integrins αvβ3 and αvβ5, was developed to disrupt tumor–ECM interactions and inhibit angiogenesis, highlighting the potential of targeting ECM‐binding receptors in cancer [202]. Additionally, interactions between ECM components and therapeutics can influence drug activity; for example, the efficacy of the anti‐angiogenic antibody bevacizumab (Avastin), which targets VEGF‐A, may be modulated by glycosaminoglycans like heparin that bind VEGF and alter its bioavailability, suggesting that matrix molecules can indirectly impact therapeutic outcomes [203]. Understanding the multifaceted roles of matrix is essential for advancing fields like cancer research, given that it is a dynamic integral component of the tumor microenvironment. Matrix contains multiple bioactive macromolecules with either tumor‐suppressing or tumor‐supporting properties, but also macromolecules that depending on their form, structure, or conformation may have opposing actions like, for example, hyaluronan (HMW versus LMW) or perlecan (intact PG versus C‐terminal endorepellin). This article has focused on specific matrix macromolecules with such dual functions, highlighting the matrix as the ultimate ‘tango partner’ of tumor cells to grow, expand, and survive aiming at the development of new matrix‐based therapeutic strategies as well as potential biomarkers for tumor diagnosis and monitoring.

## Conflict of interest

There is no conflict of interest.

## Author contributions

SSS conceived and designed the study; SSS, MEC, ET, and TDS wrote the paper.
